# Effects of postural specific sensorimotor training in patients with chronic low back pain: study protocol for randomised controlled trial

**DOI:** 10.1186/s13063-015-1104-4

**Published:** 2015-12-15

**Authors:** Michael A. McCaskey, Corina Schuster-Amft, Brigitte Wirth, Eling D. de Bruin

**Affiliations:** Department of Health Sciences and Technology, Institute of Human Movement Sciences, ETH Zurich, Wolfgang-Pauli-Strasse 27, Zurich, 8093 Switzerland; Reha Rheinfelden, Research Department, Salinenstrasse 98, Rheinfelden, 4310 Switzerland; Institute of Rehabilitation and Performance Technology, Bern University of Applied Sciences, Pestalozzistrasse 20, Burgdorf, 3400 Switzerland; Balgrist University Hospital, Forchstrasse 340, Zurich, 8008 Switzerland; Department of Epidemiology, CAPHRI School for Public Health and primary Care, PO Box 616, Maastricht, 6200 Netherlands; Maastricht University, Centre for Evidence Based Physiotherapy, PO Box 616, Maastricht, 6200 Netherlands

**Keywords:** Chronic low back pain, Sensorimotor training, Proprioception, Postural control, Functional status, Uncontrolled manifold

## Abstract

**Background:**

Sensorimotor training (SMT) is popularly applied as a preventive or rehabilitative exercise method in various sports and rehabilitation settings. Yet, there is only low-quality evidence on its effect on pain and function. This randomised controlled trial will investigate the effects of a theory-based SMT in rehabilitation of chronic (>3 months) non-specific low back pain (CNLBP) patients.

**Methods/Design:**

A pilot study with a parallel, single-blinded, randomised controlled design. Twenty adult patients referred to the clinic for CNLBP treatment will be included, randomised, and allocated to one of two groups. Each group will receive 9 x 30 minutes of standard physiotherapy (PT) treatment. The experimental group will receive an added 15 minutes of SMT. For SMT, proprioceptive postural exercises are performed on a labile platform with adjustable oscillation to provoke training effects on different entry levels. The active comparator group will perform 15 minutes of added sub-effective low-intensity endurance training. Outcomes are assessed on 4 time-points by a treatment blinded tester: eligibility assessment at baseline (BL) 2–4 days prior to intervention, pre-intervention assessment (T0), post-intervention assessment (T1), and at 4 weeks follow-up (FU). At BL, an additional healthy control group (*n* = 20) will be assessed to allow cross-sectional comparison with symptom-free participants. The main outcomes are self-reported pain (Visual Analogue Scale) and functional status (Oswestry Disability Index). For secondary analysis, postural control variables after an externally perturbed stance on a labile platform are analysed using a video-based marker tracking system and a pressure plate (sagittal joint-angle variability and centre of pressure confidence ellipse). Proprioception is measured as relative cervical joint repositioning error during a head-rotation task. Effect sizes and mixed-model MANOVA (2 groups × 4 measurements for 5 dependent variables) will be calculated.

**Discussion:**

This is the first attempt to systematically investigate effects of a theory-based sensorimotor training in patients with CNLBP. It will provide analysis of several postural segments during a dynamic task for quantitative analysis of quality and change of the task performance in relation to changes in pain and functional status.

**Trial registration:**

Trial registry number on cliniclatrials.gov is NCT02304120, first registered on 17 November 2014.

## Background

In 2006, the European Cooperation in Science and Technology working group B13 (COST B13) published guidelines for chronic non-specific lumbosacral back pain (CNLBP) treatment reporting a prevalence of CNLBP at 23 % [1]. The State Secretariat for Economic Affairs in Switzerland has released corresponding numbers in the context of preventive measures for occupational settings. According to its author, 18 % of all employees in Switzerland have reported some form of work-related back pain accounting for 26 % of occupational absence with corresponding socio-economic consequences [2]. Although the bulk of the direct costs have been attributed to care by medical physicians and non-physicians, it is the indirect costs through absenteeism and social isolation that cause more than 80 % of health costs [3]. Hence, research promoting return to normal activity and prevention of chronicity of pain remains of great importance.

CNLBP persists for more than 12 weeks and cannot be attributed to a recognisable, known specific pathology (*International Classification of Diseases* (ICD) 54.5) [1]. Lack of variable sensorimotor input has been described as a contributing factor to the development of CNLBP [4–6]. In modern society, dynamic movements are becoming ever more neglected and repetitive tasks seem to dominate most of our activities. It has been well-established that occupations requiring prolonged periods of static standing are associated with development of musculoskeletal disorders including CNLBP [7–9]. Long-term monotonous afferent input is believed to impair the sensorimotor system; circuits regulating the appropriate amount of symmetric muscle force, needed to adapt the correct posture in any given situations, are thought to be disturbed [6, 10, 11]. If not restored, this constant malfunctioning of muscular control and regulation of dynamic movement may lead to inappropriate muscular activity [11, 12] and is thought to contribute to taut muscles, imbalanced muscle activation, poor posture, and ultimately to musculoskeletal pain in lumbar regions [13].

Consequently, neuromuscular rehabilitation techniques addressing sensory deficiencies have emerged in recent years and have received increasing therapeutic attention [6, 14]. These techniques could broadly be summarised as sensorimotor training (SMT) methods aiming at increased proprioceptive input to improve motor response in dynamic environments. This might lead to improved quality of postural control, which in turn may alleviate postural specific musculoskeletal pain [15, 16].

There has been some doubt whether SMT can actually improve proprioceptive acuity in a functional way at all. In a recent review, Ashton-Miller et al. outlined a row of concerns (e.g. lack of neurophysiological evidence) about the validity of current proprioceptive exercises [17]. Although many therapists and clinicians report successful treatment cases, the exact effect and validity of sensorimotor interventions is still discussed controversially [17–19]. Despite extensive research activity on the topic of CNLBP, which has significantly contributed to the understanding of pain [20], the European guidelines on the management of CNLBP conclude that the effects of specific exercises, such as SMT, must be further evaluated [1].

The aim of this study is to compare the effects of SMT on pain and functional status with sub-effective low-intensity training (SLIT) in patients with CNLBP: Is a sensorimotor training added to physiotherapy (PT) more effective than physiotherapy with added sub-effective low-intensity training regarding pain and functional status in patients with non-specific low back pain? It is first hypothesised that functional status and self-reported pain will reduce significantly in both groups, but the SMT group will show significantly more improvement when compared to SLIT.

With novel methods available, it has become possible to quantitatively analyse the influence of pain on postural control strategies. Using the uncontrolled manifold approach [21], this study has the secondary aim to describe how much compensatory variability is being applied to maintain the postural control during perturbed stance and whether proprioceptive integration improves with SMT.

## Methods/Design

### Ethics and reporting

The study protocol follows the Consolidated Standards of Reporting Trials (CONSORT) statement on randomised trials of non-pharmacological treatment [22] and Standard Protocol Items: Recommendations for Interventional Trials (SPIRIT) guidance for protocol reporting [23]. The procedures have been approved by the local ethics committee (EC North-western Switzerland, EC number: 2014–337) and conform to the guidelines of Good Clinical Practice E6 (R1) and the Declaration of Helsinki. No data was recorded before written informed consent to participate and to publish was given by the participant.

### Study design

**Fig. 1:**
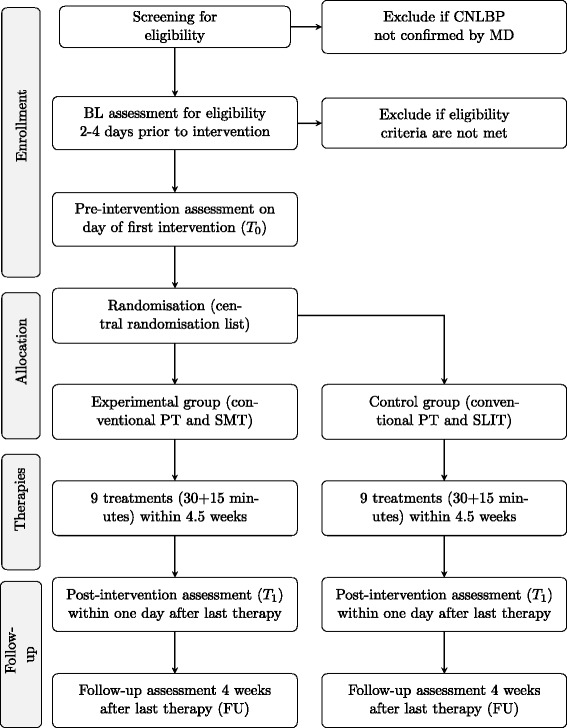
Flow chart of study procedures. BL = baseline; MD = Doctor of Medicine; PT = physiotherapy; SLIT = sub-effective low-intensity endurance training; SMT = sensorimotor training

The SensoriMotor training and Postural control in Pain rehabilitation trial (SeMoPoP) is designed as an assessor-blinded exploratory trial with 2 parallel groups and primary endpoints of pain and functional status before and after the 5-week intervention programme. Additionally, a 4-week follow-up (FU) assessment shall deliver data for intermediate-term effects. Figure 1 summarises the study design.

### Randomisation, group allocation, and allocation concealment

The randomisation list is stored with the clinic’s pharmacy, out of reach and out of sight of the investigator and all treating therapists. The list was computed-generated prior to the trial beginning by a third party, who is not involved in patient recruitment, organisation, assessment, or treatment. Mixed randomisation steps were applied using block-wise and simple-randomisation to achieve the unpredictable 1:1 allocation sequence, as has been recommended for smaller group sizes [24]. Prior to the first treatment, the responsible therapist will call the central pharmacy within the clinic to learn the patient’s group allocation. Blinding of assessors and data analysts will be maintained until study completion. During statistical analysis, the groups will be referred to without specification of treatment plan (e.g. groups A and B).

### Study population

Patients are being recruited from the outpatient department at a neurological and orthopaedic rehabilitation centre in Switzerland. Interventions, assessments and data collection, and data analysis will be conducted at the same study site. Adult patients (≥18 years) referred to the trial clinic for CNLBP treatment by their general practitioner will be invited to participate in the trial. If no medical referral has been given, e.g. as a response to the public invitation in local print media, an independent rheumatologist at the study site will examine the patient for eligibility and to confirm diagnosis (CNLBP). Symptoms included are any chronic (>3 months) pain or discomfort localised below the costal margin and above the inferior gluteal folds, with or without referred leg pain [1]. Written, informed consent must be provided prior to the beginning of any of the study procedures.

Meeting any of the following criteria will lead to exclusion [1, 25]: clinical sign of neurological damage with sensorimotor impairments (i.e. radicular syndrome, paresis or tingling in limbs); suspected or confirmed spinal pathology (e.g. tumour, infection, fracture or inflammatory disease); history of spinal surgery (e.g. decompensation or stiffening); whiplash incidence within the last 12 months; cervical pain that reduces active movement to less than 30° rotation to each side; known vestibular pathologies; major surgery scheduled during treatment or FU; physiotherapy with SMT during the last 12 weeks; inability to follow the procedures of the study: e.g. due to language problems, psychological disorders, dementia of the participant; parallel participation in another study; previous enrolment into the current study.

### Study intervention

All participating patients will attend 9 sessions of 45 minutes duration consisting of 30 minutes standard physiotherapy according to European guidelines (COST, [1]) with either added experimental (15 minutes SMT) or added control exercise (15 minutes SLIT). Sessions will take place twice a week over a 4.5-week period.

The intensity and duration of SLIT in the control group was deliberately instructed to be lower and shorter than is recommended [26]. This was used as a quasi-sham [27] to control time spent with therapist.

There is a wide variety of ways in which SMT can be performed [14, 15]. For this study, proprioceptive postural training (PPT) will be applied using the neuro-orthopaedic therapy device Posturomed (Haider Bioswing GmbH, Pullenreuth, Germany). The Posturomed consists of a labile platform, with adjustable damped swaying behaviour. Mediolateral and anteroposterior sway are increased when the two damping brakes, one at the front and one at the back, are released. This allows three specific configurations with increasing levels of instability. The Posturomed is used for therapy, but has also been used for assessment of postural control [28]. In contrast to most proprioceptive training devices, the exercise plan for PPT is clearly defined, quickly explained to the patient and easily understood [29].

Taking part in the study will not affect the patient’s prescribed treatment plan but PPT will not be part of the PT sessions. Other than that, the study protocol does not dictate the PT content or restrict any concomitant care. Detailed documentation of provided treatments will be recorded on therapy documentation sheets. Interventions are described in detail according to the Template for Intervention Description and Replication (TIDieR) guidelines [30], see Table 1 on page 5.

**Table 1:** Description of study interventions based on the Template for Intervention Description and Replication (TIDieR) checklist [30]

Item	Experimental group	Control group
1. Brief name	Sensorimotor training	Low-intensity cardiovascular training
2. Why?	Sensorimotor control is believed to be impaired in chronic non-specific low back pain. PPT is a well- defined SMT method with standardised applications. PPT is indicated for postural specific back pain, functional instability of weight-bearing joints (e.g. knee or ankle instability), hypermobility, and other postural deficiencies	Physical activity at low intensity for only 15 minutes is not expected to induce a specific treatment effect to the sensorimotor system [19] but can improve the global perception of well-being and can, therefore, be recommended as part of CNLBP treatment [61]
3. What materials?	PPT uses the Posturomed therapy device [29], which is a labile platform restricted to damped anterior-posterior and mediolateral sway. Patients will receive an exercise diary to record adherence and progress	Cardio-exercise machines: elliptical cross-trainer, treadmill, stationary bike-ergometer. Patients will receive an exercise diary to record adherence and progress.
4. What procedures?	Nine therapy sessions, each lasting 15 minutes. Therapy instructions advise seven stages of difficulty. On all stages the patient is asked to provoke oscillation by stepping on site. After 3 steps, the patient must stand still on 1 leg for 2 seconds before he or she repeats the steps. Difficulty is increased by a) decreasing the damping through release of the breaks and b) through added juggling of a ball during the motor task and trunk rotation (dual-task and divided attention). The next stage is reached once stabilisation in the previous stage is secured. The exercise is repeated for as many times as it can be performed adequately without losing balance. The moment where sensory depletion is observed by the supervising therapist, the exercise is interrupted. The exercise should be repeated for approximately 15 minutes	Nine therapy sessions, each lasting 15 minutes. Choosing either the treadmill, elliptical cross-trainer, or a stationary bike, the patient will be instructed and positioned according to body constitution. Next, patients will be asked to begin the exercise at a comfortable pace where speaking is still possible (Borg scale 6–9) and to maintain this intensity for 15 minutes
5. Who provides?	Physiotherapists trained in PPT	Physiotherapists and sport scientists
6. How?	Both intervention groups will receive initial instruction by a therapist. The patients will then perform the exercises individually with passive supervision by the therapist (e.g. promoting to next difficulty level)	
7. Where?	Both interventions will be performed in the medical training centre for physical exercise within the clinic	
8. When and how much?	During the 4.5-week intervention program, patients will receive the same allocation of 9 sessions for 15 minutes each (twice a week). This is added to the 30 minutes of conventional therapy both groups are entitled to according to their physician’s referral	
9. Tailoring	Particularly the conventional therapy will be tailored to the needs and abilities of each individual patient. The therapist may apply any form of active or passive treatment during the first 30 minutes (excluding PPT)	
	Patients will always start with the easiest level, but it is not rigorously prescribed which level they must achieve. They should try to reach sensorimotor depletion as judged by the supervising therapist (i.e. can no longer stabilise all segments at the given level of difficulty)	The low-intensity cardiovascular training is in itself tailored, as it requires each patient train at his or her individual recovery level (Borg scale 6–9).

*CNLBP* chronic non-specific low back pain, *SMT* sensorimotor training, *PPT* postural proprioceptive training

### Staff eligibility

Only a selected group of therapists at the study site will conduct the intervention. To qualify, therapists must have completed their PT training and show competences in musculoskeletal rehabilitation in patients with low back pain and PPT methods. Competences in these areas will be assumed after completion of internal workshops for the treatment under investigation, led by a certified instructor.

### Study outcomes

A treatment-blinded assessor will test patients on four measurement events (ME) (Fig. 1). The eligibility assessment at baseline (BL) will take place 2–4 days prior to intervention on the patients’ first visit to the trial site. Pre-intervention assessment (T0) will be recorded on the day of the first therapy session and post-intervention assessment (T1) within 1 day after the last intervention session at 4.5 weeks. Intermediate-term to long-term effects of the intervention will be assessed at a 4-week FU examination (FU). Apart from primary and secondary outcomes, patient characteristics will be recorded to describe the study sample (age, size, weight, activity level, occupation, other therapies, and medication).

### Primary outcomes

With the study’s primary aim to determine the effects of SMT on pain and functional status compared to usual treatment of patients with CNLBP, the primary outcomes are intended to record mean change of self-reported pain and related limitations in daily activities from T0 to T1 and T0 to FU. This is in line with recommendations by Deyo et al. for the use of standardised outcomes in clinical research on low back pain [31].

#### Functional status

Self-reported impairment in daily activities will be assessed using the German version of the Oswestry Disability Index (ODI-D), which has shown good reliability (*r* = 0.96) [32] and responsiveness [33]. The ODI consists of ten items related to daily activities (pain, body hygiene, lifting objects, walking, sitting, standing, sleeping, sexual behaviour, social life, and travelling). Each item can be rated from 0 (no pain during activity or pain getting worse) to 5 (I cannot do it myself). The total score is reported in percentage of the total achievable 50 points (from 0 % = minimal impairment to 100 % = bedridden). A change of 8 % is considered as clinically relevant [34].

#### Pain

Self-reported pain will be assessed using a German version of the Visual Analogue Scale (VAS). The VAS is a 100-mm line with 2 endpoints representing the extreme states ‘no pain’ and ‘pain as bad as it could be’. It has shown to have good re-test reliability (*r* = 0.94) with a 13-mm difference on the scale to be considered as clinically relevant [34].

### Secondary outcomes

The secondary outcomes, joint variation and postural control during perturbed stance on a labile platform, will be measured at each measure event (ME) (BL, T0, T1, and FU) using a combination of several outcome measures described below.

#### Postural control - centre of pressure

Postural control will be operationalised by measuring the deflection of centre of pressure (COP) recorded during the perturbed stance task. Several COP quantifying parameters have been suggested in the literature [35–37]. For the purpose of the study, COP 95 % confidence-ellipse area and standard-ellipse area (CEA and SEA) [38] will be analysed to use a measure of magnitude. Approximate entropy will be analysed to quantify the regularity or predictability of the time series, which has been reported to be more sensitive to small changes than magnitude alone [39]. Additionally, the amount of sway produced during the task, needed to return to a steady state of stationary stance after external perturbation of the base of support, will be reported as area under curve (AUC) of the acceleration of the labile platform.

#### Postural control - uncontrolled manifold index

To sufficiently describe and rate postural control, a more complex approach will be experimentally applied to this study. The uncontrolled manifold (UCM) analysis is an emerging computational approach to study motor synergies. It is based on the assumption that the central nervous system (CNS) does not control each degree of freedom (DOF) individually but rather selects a subspace of lower dimensionality (a manifold) that corresponds to a value of a performance variable that needs to be stabilised (i.e. centre of mass, COM). When a task is repeatedly analysed, the variance of the control variables (i.e. joint angles) across the attempts can be partitioned into two components: parallel and orthogonal to the UCM. As shown by Sholz et al. [40], the variance of the performance variable COM orthogonal to the UCM is usually smaller as compared to the variance parallel to it when standing in response to surface perturbation. In other words, the CNS allows relatively high variability of control variables (joint angles) as long as this variability does not cause the COM to move further away from its steady state prior to perturbation. Basically, a UCM spans a subspace consisting of all joint angles that support fast return to stability. Joint angle configurations that lie orthogonal to the UCM lead to a deviation from this stable condition and, therefore, affect the controlled variable. The relation of both subspace values to one another will be reported as the UCM-Index. For detailed description of the application refer to Scholz et al. [21].

#### Proprioception

To assess conscious proprioceptive acuity, cervical joint repositioning error (C-JRE) will be measured. C-JRE is defined here as the relative error of a blindfolded replication of a verbally instructed head position at 0°, 30°, and 60° in the horizontal plane [41, 42].

#### Measurement set-up

**Fig. 2:**
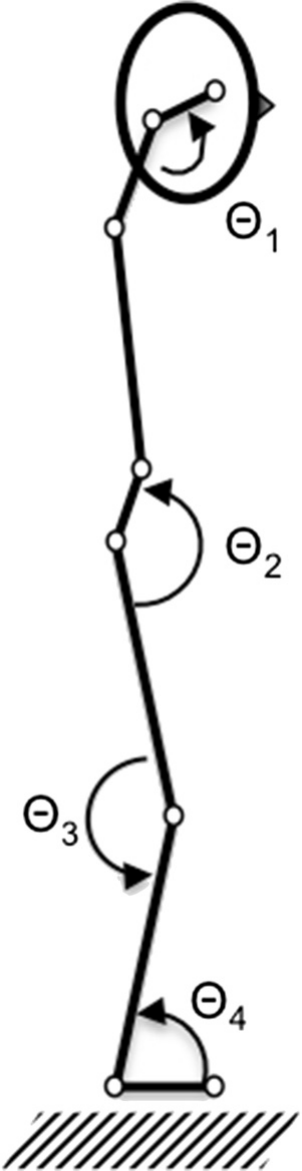
Marker configuration. Θ_1_ = Cervical angle; Θ_2_ = Hip angle; Θ_3_ = Knee angle; Θ_4_ = Ankle angle; marker positions (from head to toe): corner of the eye (orbital process of the zygomatic bone), mastoid process of temporal bone, acromion, anterior superior iliac spine, greater trochanter, lateral condyle of femur, lateral malleolus, calcaneal tuberosity, first metatarsal bone

The experimental set-up consists of a labile platform (Posturomed 202, Haider Bioswing, Pullenreuth, Germany), an attached provocation module with manual 3-cm deflection (Haider Bioswing GmbH, Pullenreuth, Germany), 2 high-speed cameras (Basler acA165-uc –-Basler AG, Ahrensburg, Germany), a personal computer with a Windows 8 (Microsoft Inc., Redmond, WA, USA) operating system to which both cameras are attached over a USB3.0 cable, a motion analysis software (Templo v.8.2, Contemplas GmbH, Kempten, Germany), an accelerometer attached to the base plate of the Posturomed, and optical markers to measure segmental joint angle variation and joint motion. For the C-JRE task, a custom 1-size lightweight helmet with an attached laser pointer and retro-reflecting markers was developed. For each head position (−60°, −30°, 0°, +30°, +60° rotation) a vertical mark is fixed to a screen facing the subject. Video-based analysis of the joint-angle and marker-position deviation will be conducted. The camera will track the reflecting markers with a spatial resolution of 1024 x 760 pixels and a temporal resolution of 100 frames per second, which has shown to suffice for the purposes of the analyses [43]. The algorithms to track the 15.9-mm reflective markers are included in the motion analysis software. Marker configuration follows the proposed scheme by Scholz et al. [40] with nine sagittal markers (Fig. 2) and additional two frontal markers to record shoulder girdle and hip girdle lateral flexion and mediolateral translation: at the corner of the eye, the mastoid process, shoulder (acromion), hip (greater trochanter and anterior superior iliac spine), knee (lateral femoral condyle), ankle (lateral malleolus), toe, heel and the platform surface. For calibration purposes, fixed geometrical objects with known metrics and fixed angles will be placed onto the labile platform and recorded in the frontal and sagittal plane. Coordinate data of each reflective marker will be filtred at 5 Hz using a bi-directional, second-order, Butterworth digital filter in Matlab^TM^ version R2014b (Mathworks Inc., Natick, MA, USA) [21]. Finally, COP is recorded using the zebris FDM-S pressure plate (60 Hz) (zebris Medical GmbH, Isny im Allgäu, Germany), which is placed on top of the swaying platform. All final analysis algorithms will be implemented and executed in Matlab^TM^ version 2014b for Mac (Mathworks Inc., Natick, MA, USA).

#### Measurement procedures

After interviewing the patient for primary outcomes with the described questionnaires, C-JRE will be tested in a seated position. Wearing a visual deprivation mask and the laser-pointer helmet, the participants will then be verbally instructed to rotate their head slowly. Using the position of the laser on the prepared screen with the marked angles, the assessor guides the participant to reach the target position. This position is held for 15 seconds and subsequently replicated 5 times. Participants are instructed to push a button fixed to the chair when they feel confident to have reached the original position. This will set a marker at the time point during the recording where the participant felt closest to the initially instructed position and is repeated for every angle. The sequence of angles is randomised prior to the test.

To assess postural control, the patient will first be asked to stand on the platform to familiarise themselves with the surface’s behaviour before the platform will be fixed to its deflected position (3 cm posterior). Then the patient will be instructed to adopt an upright posture with arms folded across the chest. On the cue ‘ready-steady-go’, the assessor will release the platform from its deflection. Two familiarisation trials will be performed prior to measurement. The swaying will be recorded in sync with the COP and video for 10 seconds (3 seconds prior to pertubation and 7 seconds after perturbation) and repeated 5 times. All of the device’s damping brakes will be released for this test to allow maximal sway and provoke the postural control response.

The setup was pilot-tested in order to define optimal settings for the recording (e.g. light, camera distance, marker-repositioning). Each ME will be of approximately 1 hour in duration.

### Sample size

Only few studies have investigated the effects of sensorimotor training on pain and functional status. Two of the most recent of these have found significant time and group interactions using the same outcomes. Applying the results of these studies to a sample size calculation with an alpha value of 5 % and the desired power 80 %, it is expected that 10 patients per arm would suffice to reveal group differences and to detect change.

Considering these findings and taking into account the explorative approach of this trial, a total number of 40 to 50 participants is planned, including 20 healthy controls for BL-comparison.

### Statistical analyses

Baseline comparability of both groups will be inspected for primary and secondary parameters. Mean change and standard deviation of change will be reported as well as mean values of each outcome at every ME. If normally distributed, a mixed (2) Group x (2) ME multivariate analysis of variance (MANOVA) with repeated measures during ME will be conducted for 6 dependent variables (Pain VAS, Functional Status ODI, Postural Control as SEA, AUC, and UCM-Index), and proprioception (C-JRE)). Non-normal distributed data will be analysed with non-parametric methods.

Intention-to-treat analyses will be performed. If necessary, an additional per protocol analysis will be carried out. Recorded outcome data of patients who drop out after inclusion will be included in the final analysis (missing data reconstructed based on mean changes and standard deviations of the other participants of the group).

## Discussion

The primary aim of this study is to pilot-test a study design and measurement setup to evaluate the efficacy of SMT in the rehabilitation of musculoskeletal pain. As shown in a recent systematic review [14], the justification of SMT in pain rehabilitation is highly questionable from an evidence-based perspective. Moreover, there are no recommendations for dose, frequency or intensity of SMT at which training effects could be expected [14, 26]. With no standardised recommendations regarding its implementation, SMT studies present large practical heterogeneity and can barely be compared systematically. Hence, it remains a vastly under-examined intervention for pain rehabilitation. There is still need for clinical parameters that are sensitive enough to capture small changes in movement behaviour that are expected to improve after or are indicative for SMT.

As the aim of chronic pain treatment should be to make the patient feel better and increase quality of life, from a clinical perspective, the most important outcome is the self-reported pain and functional limitations in daily activities. If these outcomes do not improve, the treatment should be adapted to tackle other modalities of pain development. Moreover, pain may change irrespective of the change of the aspect of physical functioning targeted by the intervention (i.e. sensorimotor control) [44]. However, to make a clinically informed decision, it is necessary to know whether symptom development is related to the intervention receiving most attention during the therapy.

One challenge in SMT evaluation lies in the choice of an appropriate parameter to measure change over time or compare different populations. Change could occur at any one level of the complex neural pathway involved in the regulation of peripheral sensory integration. In previous low back pain-related intervention studies, SMT has been assessed using joint repositioning sense [41, 42, 45], COP for postural sway [46, 47] and neurophysiological measures [48]. While JRE only tests one aspect of a single joint involved in the muscle chain of postural control, COP only tests the summation of all joints involved and cannot be dissected to show each segment’s contribution [49]. In a reductionist approach, neurophysiological assessments allow investigation of the functions, or dysfunctions, of key elements involved in any given task (e.g. evoked potentials or synaptic activities at neuromuscular junctions and their pathways). Accumulated information of these elements is used to interpret the overlying, more complex system [50]. However, as pointed out by Latash et al., the ‘function of a complex system cannot be understood through its structure and the properties of its elements’ [50]. Following this notion, one of this study’s purposes is not to analyse the effect of chronic pain on individual elements, but rather to examine the dynamic strategies adopted by patients with CNLBP to integrate sensory input when controlling COM under perturbed stance conditions.

The relationship of pain and changes in motor control has been shown in several studies [51–56] and is seen as a protective reaction of the body to limit provocation of the painful area [57]. The proposed dynamic analysis setup with anticipated perturbation will allow investigation of segmental behaviour at any given moment during stance and allow description of each segment’s contribution to the control of COM when recovering stability. This is an important aspect to not only understand the variability of postural control observed when comparing CNLBP patients with a pain-free population sample, but also to explain within-group variability of COM parameters. Understanding this aspect may help to better target faulty movement strategies and describe its part in underlying mechanisms of pain development.

A challenging limitation of the presented study is going to be the interpretation of the magnitude of variability. Being a relatively young field of research within human kinetics, there are not sufficient findings to describe the optimal amount of variability needed to maintain healthy posture [36, 39, 43]. It is yet to be elicited which degree of variability is necessary to remain adaptive toward external and internal perturbations and at which threshold variability causes deviation from the task’s individual goal [39]. Relating joint configurations parallel to the manifold and joint configuration perpetual to the manifold (i.e. UCM-Index) offers a potential evaluation of individual movement quality. As the former causes the COM to return to the point before perturbation while the latter describes the amount of joint configurations causing deflection from initial COM position, a high index would be desirable. Provided the setup proves to be robust enough to record sensitive changes after postural specific SMT and differences across population, a large-scale clinical trial with large sample sizes for both groups could be conducted to identify optimal levels of both components during postural control. This would also allow subgroup-analysis; it is widely accepted that subgroups of patients with CNLBP exist, e.g. with or without movement control impairments [58] or with different risk profiles [59]. The population included may have a variety of different causes for their pain. Hence, function or postural control will not necessarily improve when pain does and vice versa. However, due to its explorative nature, this trial has a limited sample size which would not allow subgrouping [60].

A general limitation to therapeutic trials involving exercise is the limited possibilities to blind the patients from knowing the experimental arm. This is particularly problematic in studies where subjective pain measures are evaluated. To reduce the risk of detection bias, all assessors and data analysts will be blinded to the intervention allocation.

To allow comparability of the conventional PT, a detailed documentation of all exercises and treatment applied during a session will be recorded. However, as the study includes outpatients, it is not controlled regarding what kind of leisurely activities and possible exercises are conducted. In this sense, co-interventions cannot be controlled.

If proven feasible and effective, the study will provide an objective, quantifiable and sensitive clinical assessments and a standardised procedure for SMT to implement in a large-scale study.

## Trial status

The trial is currently recruiting patients and will require 12–14 months to complete all follow-up assessments.

## Abbreviations

AUC: area under curve
BL: baseline assessment
C-JRE: cervical joint repositioning error
CEA: confidence-ellipse area
CNLBP: chronic non-specific low back pain
CNS: central nervous system
COM: centre of mass
CONSORT: Consolidated Items of Reporting Trials
COP: centre of pressure
DOF: degrees of freedom
EC: Ethics Committee
FU: follow-up assessment
ICD: *International Classification of Diseases*
MANOVA: multivariate analysis of variance
ME: measurement event
ODI: Oswestry Disability Index
PPT: proprioceptive postural training
PT: physiotherapy
SEA: standard-ellipse area
SeMoPoP: SensoriMotor training and Postural control in Pain rehabilitation
SLIT: sub-effective low-intensity training
SMT: sensorimotor training
SPIRIT: Standard Protocol Items: Recommendations for Interventional Trials
T0: pre-intervention assessment
T1: post-intervention assessment
TIDieR: Template for Intervention Description and Replication
UCM: uncontrolled manifold
VAS: visual analogue scale
